# Spectral Domain OCT: An Aid to Diagnosis and Surgical Planning of Retinal Detachments

**DOI:** 10.1155/2011/725362

**Published:** 2011-12-29

**Authors:** Graham Auger, Stephen Winder

**Affiliations:** Department of Ophthalmology, Royal Hallamshire Hospital, Glossop Road, South Yorkshire, Sheffield S10 2JF, UK

## Abstract

Regmatogenous retinal detachments need prompt intervention particularly when macula is on. Unfortunately this is not always easy to ascertain clinically and the chronicity of the event is often muddled in patient's histories. Developments in optical coherence tomography (OCT) have allowed high-resolution axial scans which have enabled the characterisation of retinal changes in retinal detachments. In this paper, we show the changes in retinal morphology observed by spectral domain OCT and how this can be used to plan appropriate surgical intervention.

## 1. Introduction

Rhegmatogenous retinal detachments referred in an acute nature require prompt surgical repair. However, studies have shown that surgery is best done during normal working hours [1]. Given the pressures on theatre use it is important to be able to assess the retinal detachment and to ascertain the urgency of planning surgical intervention. One of the most important features is the involvement of the macula and fovea that is macula on or macula off. In cases of macula-off retinal detachments, visual outcome is less dependent on prompt surgery and surgical correction can be delayed [1]. Macula-on retinal detachments, however, should have their surgery expedited, the main concern being the conversion to a macula-off situation which has a much poorer visual prognosis [1].

The assessment of rhegmatogenous retinal detachments is multifactorial; in an otherwise normal eye visual acuity is an easy measure of macula involvement with the 6/60 patient being macula off and 6/6 macula on [1]. Similarly the onset of symptoms and the age of the retinal detachment is important, as chronic detachments can be more stable and surgery can be safely delayed [1]. Also the extent of detachment and position of the retinal break can also help predict the progression of an acute macula on retinal detachment [1]. However, in certain situations the macula-on or macula-off question is not easily answered; visual acuities may be misleading; examination of the detachment may be difficult due to poor views often due to vitreous hemorrhage and chronicity may be difficult to ascertain in patients with vague histories.

High-speed spectral domain optical coherence tomography (OCT) offers a noninvasive tool to evaluate retinal microstructural changes in a number of eye pathologies. Newer systems using spectral domain calculations have improved data acquisition speeds compared with conventional time-domain OCT equipment allowing much greater axial resolution [2]. Given the greater resolution a number of characteristic changes seen in retinal detachment have been observed. In this paper, we discuss two cases where spectral domain OCT and an understanding of the histological changes have enabled a clearer diagnosis and planning of treatment.

## 2. Case 1

**Figure 1:**
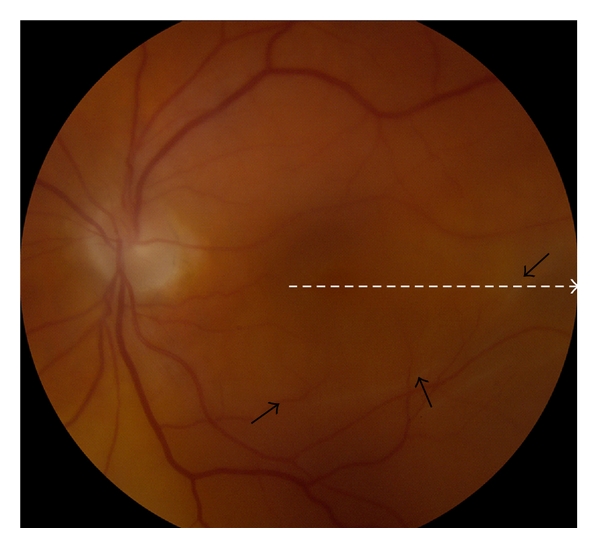
Colour fundus photograph of retinal detachment secondary to an inferior temporal retinal tear. Black arrows indicate the initially suspected margin of the retinal detachment. Dotted line depicts the direction of the OCT scan shown in Figure 2.

Our first case is a seventy-five-year-old gentleman who presented with a vague history of blurred vision for six weeks. Visual acuity was 6/24 and examination revealed a pseudophakic inferotemporal macula-off retinal detachment. The reduction in visual acuity was thought to be secondary to vitreous haemorrhage as biomicroscopy assessment showed the detachment stopping inferior to the macula (Figure 1).

**Figure 2:**
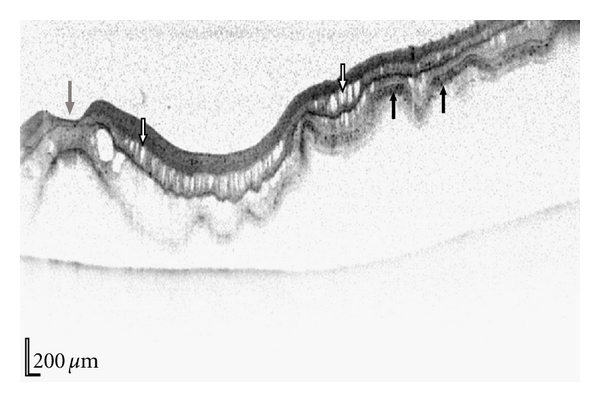
Horizontal spectralis OCT of the retinal detachment shown in Figure 1, scan direction is indicated by the white dotted line in Figure 1. Characteristic changes seen on OCT in retinal detachments are observed including retinal folds, intraretinal cysts (white arrows), and hyperreflectivity of the photoreceptor layer (black arrows). Fovea is denoted by a grey arrow.

**Figure 3:**
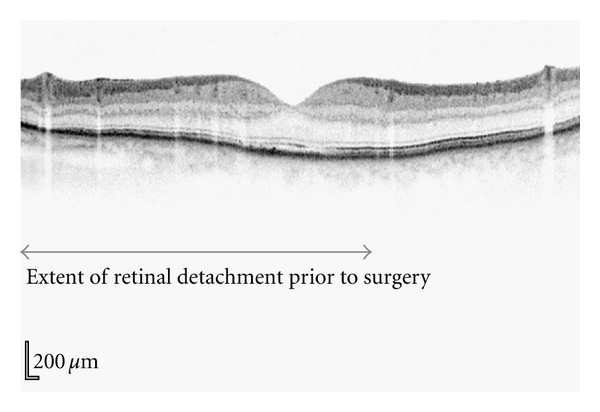
Postsurgical vertical OCT of the retinal detachment shown in Figures 1 and 2. The area of retinal detachment prior to surgery is represented by the bar below the OCT. Restoration of normal morphology has occurred one year following retinal detachment repair.

To confirm the macula status, a microstructural imaging analysis was performed using the Heidelberg Spectralis OCT scanner. Contrary to the biomicroscopy examination (Figure 1), this revealed a macula-off retinal detachment (Figure 2). Changes seen in the OCT scan were characteristic of an old retinal detachment with the presence of intraretinal cysts, undulation of outer retinal layers, and the hyper-reflectivity in the photoreceptor layer (Figure 2). Secondary to these OCT findings, the surgical session was deprioritised and performed five days later. The surgical repair consisted of a three-port pars plana vitrectomy with perfluoropropane tamponade and cryotherapy. Postoperative visual outcome was good being 6/9 two months after surgery. Subsequent spectralis OCT one year following the retinal detachment shows restoration of normal retinal morphology with resolution of the intraretinal cysts, flattening of the retinal layers, and no hyperreflectivity seen (Figure 3).

## 3. Case 2

**Figure 4:**
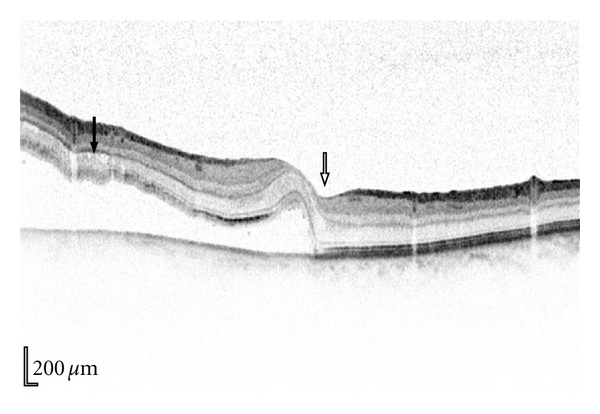
Acute retinal detachment that transects the fovea (white arrow), cystic changes are present peripherally (black arrow) but the fovea remains morphologically intact although shallowly detached from the pigmented epithelium.

Our next case was a fifty-year-old myopic female who presented on a Friday with a several-month history of floaters and visual distortion described as “looking through Vaseline.” Visual acuity was reduced to 6/12. Biomicroscopic examination showed an inferior macula-off retinal detachment. Microstructural analysis of the macula was performed using a Heidelberg Spectralis OCT scan which confirmed a macula-off retinal detachment; however, the OCT scan revealed that the fovea was bisected by this detachment (Figure 4). Moreover, the macula microstructure seen in the OCT scan showed no retinal folds or hyperreflectivity present near the fovea. Indeed, the only morphological retinal detachment changes observed which indicated any chronicity were small intraretinal cysts present peripherally away from the fovea (Figure 4).

Given the OCT findings, she was treated as a macula-on retinal detachment patient, and surgery was expedited such that an emergency theatre session was organised within 24 hours on a Saturday morning. The surgical repair was a three-port pars plana vitrectomy using a sulphur hexaflouride tamponade and cryotherapy. After subsequent cataract surgery, vision had returned to 6/6 with normal OCT findings (scan not shown).

## 4. Discussion

The morphological changes seen in retinal detachment have previously been evaluated by OCT and are becoming clearer with newer systems using spectral domain calculations, which have improved data acquisition speeds to ~40 000 A-scans per second allowing much greater axial resolution to approximately 3.5 *μ*m tissue resolution [2]. The transformations seen in retinal detachment include intraretinal cyst formation, intraretinal separation, and undulation of outer retinal layers [3, 4]. The disruption of the photoreceptor inner and outer segment junction in macula-off rhegmatogenous retinal detachments is also seen both preoperatively [5] and postoperatively [6, 7]. Murine models comparing histology and OCT confirm these findings and also highlight the hyperreflectivity in the photoreceptor layer which may represent a cellular immune infiltration or misalignment of the photoreceptor layer [8]. These changes were all seen in our first case (Figure 2) proving that the retinal detachment had been present for a period of time prior to arrival in our unit and enabling appropriate de-prioritisation within a busy vitreoretinal service.

In our second case, in which the fovea was bisected by a retinal detachment, time of onset was in some doubt. Retinal thickness of the detached retina has been shown to be time dependent initially thickening then thinning with time [8, 9]; however the subfovea thickness was normal when scanned suggesting a recent event along with the absence of any intraretinal cysts, retinal undulations, and hyper reflectivity of the photoreceptor layer (Figure 4). Onset of retinal detachment is of importance, as experimental retinal detachments in cats have shown that although alterations in the outer nuclear layer occur after 1 hour, progressive loss of photoreceptors continues up to 13–30 days [10], with limited atrophy in cat retinas detached 3 to 7 days [11]. Macular involvement in retinal detachment has a bad prognosis for visual outcome [1]. However, patients that have no tomographic structural changes presumably due to recent foveal involvement have better clinical prognosis [4]. This is most likely secondary to less atrophy and death of the photoreceptors which has histopathologically been shown to be present in prolonged detachment of the retina [10–14]. Finally, the height of retinal detachment, which appears to affect the formation of multiple cystic cavities in the detached inner and outer neuronal layers, correlates with poor visual outcome [15, 16]. All of these features when taken into account suggested a good prognostic outcome for our second patient and hence prompt surgery resulting in an excellent visual recovery; an outcome that could have been considerably poorer if surgery had been delayed and fovea atrophy had occurred.

The ability to predict the outcome of operations obviously helps plan surgery. The morphological changes in retinal detachment seen in OCT scans give prognostic factors pertaining to visual outcome and thus help anticipate surgical outcomes. This paper has shown the two scenarios where surgical prioritisation is reversed, that is, from macula on to macula off and secondly, from macula off to macula on. In our first case, a chronic detachment was identified by OCT and allowed planning within the department for higher priority operations to take place. Conversely, the lack of subfoveal morphological changes in our second case led to the conclusion that the detachment was recent and prognosis good, thus surgery was expedited. Both cases highlight the superiority of OCT imaging against biomicroscopy. We suggest that if any doubt regarding the status of the macula exists, a routine noninvasive OCT should be performed to help clarify the situation prior to surgery.

## References

[1] Ross WH (2002). Visual recovery after macula-off retinal detachment. *Eye*.

[2] Alam S, Zawadzki RJ, Choi S (2006). Clinical application of rapid serial fourier-domain optical coherence tomography for macular imaging. *Ophthalmology*.

[3] Hagimura N, Suto K, Iida T, Kishi S (2000). Optical coherence tomography of the neurosensory retina in rhegmatogenous retinal detachment. *American Journal of Ophthalmology*.

[4] Lee SY, Joe SG, Kim JG, Chung H, Yoon YH (2008). Optical coherence tomography evaluation of detached macula from rhegmatogenous retinal detachment and central serous chorioretinopathy. *American Journal of Ophthalmology*.

[5] Nakanishi H, Hangai M, Unoki N (2009). Spectral-domain optical coherence tomography imaging of the detached macula in rhegmatogenous retinal detachment. *Retina*.

[6] Smith AJ, Telander DG, Zawadzki RJ (2008). High-resolution fourier-domain optical coherence tomography and microperimetric findings after macula-off retinal detachment repair. *Ophthalmology*.

[7] Wakabayashi T, Oshima Y, Fujimoto H (2009). Foveal microstructure and visual acuity after retinal detachment repair. Imaging analysis by fourier-domain optical coherence tomography. *Ophthalmology*.

[8] Cebulla CM, Ruggeri M, Murray TG, Feuer WJ, Hernandez E (2010). Spectral domain optical coherence tomography in a murine retinal detachment model. *Experimental Eye Research*.

[9] Yetik H, Guzel H, Ozkan S (2004). Structural features of attached retina in rhegmatogenous retinal detachments. *Retina*.

[10] Barr CC (1990). The histopathology of successful retinal reattachment. *Retina*.

[11] Anderson DH, Guerin CJ, Erickson PA (1986). Morphological recovery in the reattached retina. *Investigative Ophthalmology and Visual Science*.

[12] Erickson PA, Fisher SK, Anderson DH (1983). Retinal detachment in the cat: the outer nuclear and outer plexiform layers. *Investigative Ophthalmology and Visual Science*.

[13] Wilson DJ, Green WR (1987). Histopathologic study of the effect of retinal detachment surgery on 49 eyes obtained post mortem. *American Journal of Ophthalmology*.

[14] Lai WW, Leung GYO, Chan CWS, Yeung IYL, Wong D (2010). Simultaneous spectral domain OCT and fundus autofluorescence imaging of the macula and microperimetric correspondence after successful repair of rhegmatogenous retinal detachment. *British Journal of Ophthalmology*.

[15] Lecleire-Collet A, Muraine M, Ménard JF, Brasseur G (2006). Evaluation of macular changes before and after successful retinal detachment surgery using stratus-optical coherence tomography. *American Journal of Ophthalmology*.

[16] Schocket LS, Witkin AJ, Fujimoto JG (2006). Ultrahigh-resolution optical coherence tomography in patients with decreased visual acuity after retinal detachment repair. *Ophthalmology*.

